# A 19 year population-based cohort study analysing reoperation for recurrence following laparoscopic and open inguinal hernia repairs

**DOI:** 10.1007/s10029-019-02073-w

**Published:** 2019-11-30

**Authors:** G. Ramsay, N. W. Scott, J. O. Jansen

**Affiliations:** 1 Rowett Institute, University of Aberdeen, Ashgrove Road West, Aberdeen, AB25 2ZD UK; 2 Medical Statistics Team, Division of Applied Health Sciences, Polwarth Building University of Aberdeen Foresterhill, Aberdeen, AB25 2ZD UK; 3 Division of Acute Care Surgery, University of Alabama at Birmingham, Birmingham, USA

## Abstract

**Introduction:**

Laparoscopic (LHR) and open (OHR) inguinal hernia repairs are both used to treat primary herniae. This study analyses the rates of operation for recurrence after laparoscopic and open inguinal hernia repair, at a population level, while considering competing risks, such as death and other operative interventions.

**Methods:**

This is a population cohort study in Scotland. All adult patients who had a primary inguinal hernia repair in Scotland between 01/04/1996 and 01/01/2015 were included. The main outcome was recurrent operations. Cumulative incidence functions (CIF) were calculated for competing risks of death. A cox proportional hazards regression model was used to control for confounders of age, gender, bilateral herniae, deprivation and year of procedure.

**Results:**

Of 88,590 patients, there were 10,145 LHR and 78,445 OHR. Recurrent operations were required in 1397 (1.8%) OHR and 362 (3.6%). LHR had greater hazard of recurrence than OHR (HR 1.83, 95% CI 1.61–2.08, *p* < 0.001). Faster time to recurrence was also associated with being older (HR for one year increase: 1.010, 95% CI 1.007–1.013, *p* < 0.001), being more affluent (HR 1.18, 95% CI 1.01–1.38, *p* = 0.04) and having a bilateral index operation (HR 2.53, 95% CI 2.22–2.88, *p* < 0.001).

**Conclusions:**

LHR is becoming more popular in Scotland over the past 2 decades. However, when other key confounding factors are controlled, it is associated with a higher recurrence rate.

## Introduction

Inguinal herniae are common, with an estimated prevalence of 1.7% across all ages and 4% in people over the age of 45 [1]. The lifetime risk of developing an inguinal hernia is 27% in males and 3% in females [2]. Herniation may be complicated by gastrointestinal obstruction and strangulation of abdominal viscera, and can be a source of ongoing pain [1]. Elective inguinal hernia repair aims to prevent these potential complications and remains one of the most commonly undertaken operations in general surgery [3].

For several decades, the mainstay of treatment was an “open” repair, using a prosthetic (often polypropylene) mesh [4] with reduced risk of recurrence when compared to an open suture repair [5]. In the early 90 s, minimally invasive approaches were introduced [6, 7]. Since then, laparoscopic repair has become increasingly utilised [8]. Advocates of the laparoscopic repair describe reduced incision size, reduced acute pain and a faster time to recovery, with similar complication rates to open repairs [9–13]. However, open hernia repair remains a commonly performed procedure. Unlike operations such as cholecystectomy, the laparoscopic technique has not superseded open operations in terms of numbers being undertaken [14].

Recurrence rates [15–18] and serious intraoperative complications [3, 19] have, in some studies, been found to be higher following laparoscopic than open operations, some of which have been attributed to the early implementation [17] or lack of standardisation of laparoscopic techniques [20]. Recurrence is a key outcome in hernia surgery, and usually requires re-operation. Such re-operations are also associated with worse outcomes than primary repair [27–29].

The two techniques have been mostly compared using case–control studies and randomised controlled trials [18, 24, 25]. An early trial [18] showed a recurrence rate of 10.1% in laparoscopic repairs versus 5% for open operations at 2 years. However, studies with a longer follow-up time, recurrence rates for laparoscopic and open repair have been shown to be 3.8% and 3.0%, respectively, at 4 years [25], and 4.1% and 4.9% at 7 years [19]. As these timeframes are relatively short, it is conceivable that any potential differences in outcomes between the two techniques may only become apparent later than most randomised trials’ follow-up periods. Indeed, one study that reported 20 year outcomes of a small randomised controlled trial comparing the two techniques showed a significantly higher recurrence rate in the laparoscopic group (25.7% versus 9.7%)[17]. Meta-analyses have been inconsistent in their conclusions with some showing an increased recurrence risk with laparoscopic procedures [15] and others showing comparable results to open repairs [26, 27]. Thus, the outcome profile of laparoscopic in comparison to open inguinal hernia operations remains unclear nearly 3 decades after its introduction.

There are few longitudinal studies comparing the need for surgery for recurrence between the two techniques and those that are available utilized data that are now historical [16, 28]. Longitudinal assessments require careful consideration of competing risks. For example, mortality will clearly prevent the potential for a recurrent hernia repair. Such competing risks are increasingly recognised as an important methodological concern in the surgical literature [29]. In this study, the risks of recurrent inguinal hernia repair requiring re-operation, following initial open and laparoscopic surgery, over a 19 year timeframe, using a population-based longitudinal data set, after adjusting for covariates and accounting for competing risks were compared.

## Methods

### Design, setting, and data source

This is a longitudinal cohort study. Data were obtained from the Information Services Division (ISD) of the National Health Service (NHS) in Scotland, which collects hospital episode data for the whole of Scotland. Every patient has a unique identifier, which allows their medical history to be tracked over time, even if admitted to another hospital. Anonymised individual patient data were obtained including operation type, diagnosis, further hospital admission and demographic data [age, gender and postcode-derived socioeconomic deprivation indices, expressed as Scottish Index of Multiple Deprivation (SIMD) quintiles].

### Case definitions

Information Services Division records are coded using the International Statistical Classification of Diseases and related health problems version 10 (ICD-10) system for diagnoses and the Office of Population Censuses and Surveys Classification of Surgical Operations and Procedures 4th revision (OPCS-4) for operations. All coders are trained in Scottish Clinical Coding standards.

The index procedures for this study comprised any operation for primary inguinal hernia conducted in Scotland between 1/4/96 and 1/1/15. The ICD-10 codes K40.2 (Bilateral Herniae without obstruction or gangrene) and K40.9 (Unilateral Hernia without obstruction or gangrene), admitted and operated in an elective manner were included. Coding of the laterality of the hernia (left or right) was not available for this cohort. Recurrent repairs, patients aged less than 18 years, emergency procedures and patients whose initial operation was recorded as being associated with gangrene or obstruction were excluded. Any inguinal hernia reoperation subsequent to these index procedures was defined as a recurrent repair.

The surgical approach was defined by OPCS 4 code T20 (primary repair of inguinal hernia): T202 (primary repair of inguinal hernia using insert of prosthetic material) or T209 (unspecified primary repair of inguinal hernia) were included. The codes Y75 (Minimal Access to abdominal cavity), Y751 (Laparoscopically assisted approach to abdominal cavity), Y752 (Laparoscopic approach to abdominal cavity REC) or Y76.3 (Endoscopic approach to other body cavity) were used to define a laparoscopic procedure. Those without these codes were assumed to be open. Conversion to open procedure was identified by the Y71.4 code (failed minimal access approach converted to open), and treated as open procedures.

### End points

The primary end-point was re-operation for recurrent inguinal hernia, electively or as an emergency. This was defined as an OPCS4 procedure code for further ipsilateral inguinal hernia repair (OPCS4 codes T21x). (If the initial operation had been a bilateral repair, then a further operation on either side was included as a recurrence).

### Statistical methods

Analyses were conducted using Stata^®^ (StataCorp LLC, College Station, Texas, USA) version 15. Continuous variables were described using mean and standard deviation (SD) or median and interquartile range (IQR) depending on the distribution of the data. Categorical variables were described using frequencies and percentages.

A time-to-event analysis approach was used where the event of interest was having an operation for recurrent inguinal hernia. There were two other types of events that had to be considered: (1) mortality needed to be regarded as a competing event; (2) those receiving operations for contralateral primary hernia repair (because of the structure of the Scottish database). If two primary operations are conducted it is not possible to accurately determine if a subsequent reoperation relates to the original (index) procedure or the later operation on the other side. This was due to the non-recording of laterality of hernia operations in this data set. Non-recurrent (primary contralateral) hernia operations were therefore treated as censored in the analysis since the recurrence status of the index operation thereafter becomes unknown. Patients receiving a bilateral hernia operation as part of the index procedure were, however, included in the analysis as all subsequent reoperations must be related to the index procedure.

Kaplan–Meier survival graphs are not recommended in the presence of competing risks as they do not consider possible dependence between these risks. Instead, it is considered more appropriate to present the cumulative incidence function (CIF) [30]. There are two main approaches to the analysis of competing risks when adjustment for confounders is required. For prognostic research questions, where the main aim is to calculate survival probabilities, the sub-distribution hazards approach of Fine and Gray [31] is recommended. When the aim is to investigate the magnitude of an effect size (hazard ratio) the multivariate cause-specific proportional hazards model is generally indicated [30]. The latter approach was chosen and implemented using a standard Cox proportional hazards regression model predicting time to reoperation with censoring for both mortality and non-recurrent hernia operations. Results are presented using hazard ratios (HR) with 95% confidence intervals (CI). Two similar models predicting time to mortality and non-recurrent hernia operations were also conducted. In addition, CIFs for all three analyses by type of operation were produced using the *stcrreg* procedure in Stata. In this approach, anyone experiencing a competing event is considered to be technically at risk throughout the study period.

As the type of operation received could be related to the characteristics of the patient, it is important to control for potential confounding factors. All analyses were adjusted for age, gender, Sottish Index of Multiple Deprivation (SIMD) quintile, bilateral status of the index operation and year of procedure (expressed as years since 1996).

### Approvals

This project was reviewed by the Public Benefit and Privacy Panel (PBPP) of the Information Governance division of the NHS Scotland and approved. Data were obtained through and analysed on the secure Data “Safe Haven” in the University of Aberdeen. The project was funded with NHS Grampian endowment funds.

## Results

### Baseline characteristics

**Fig. 1:**
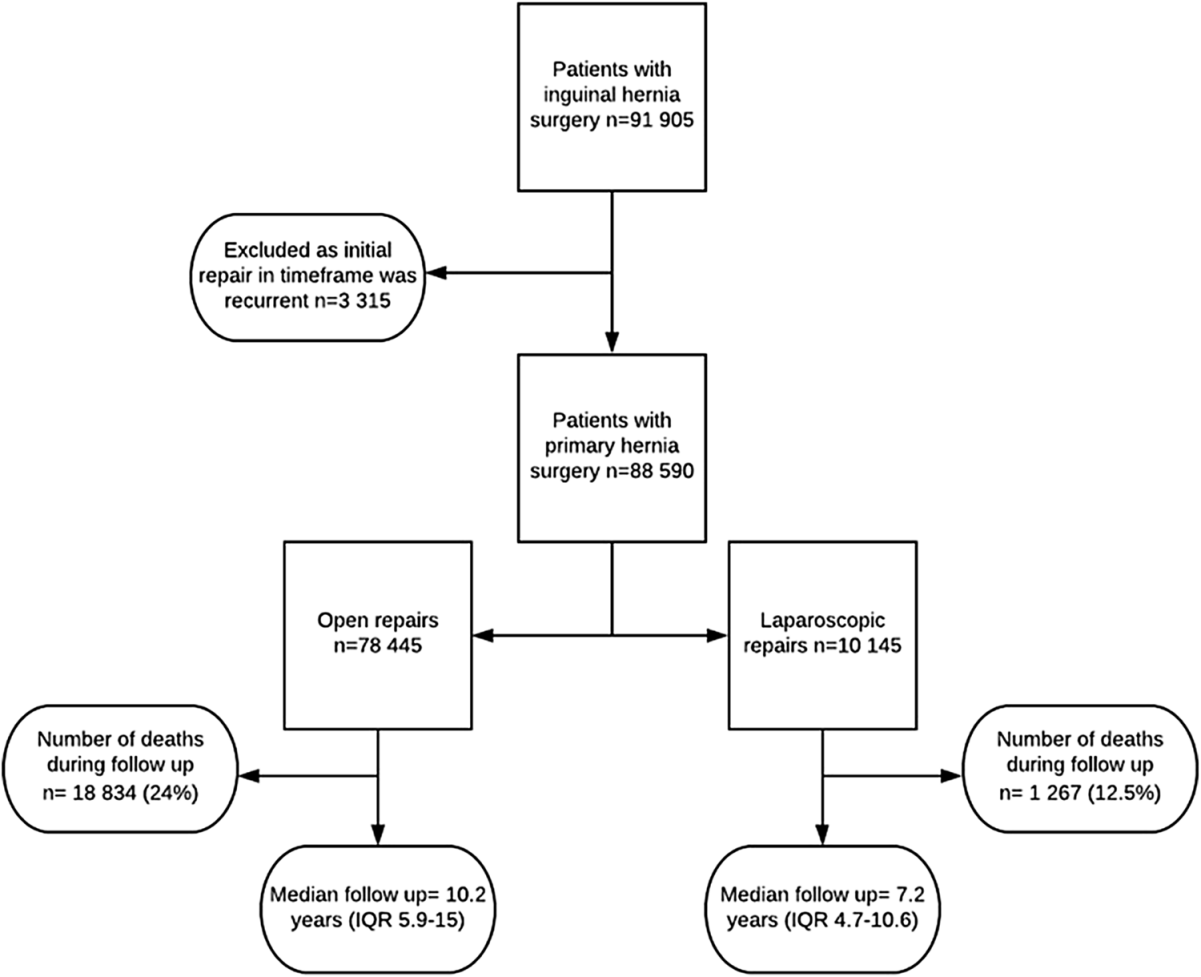
Flow chart of the patient cohort and follow-up

Over the 19 year time period, 91 905 patients had inguinal hernia surgery. Of these patients, 3315 were excluded because their initial surgery during the study period was a recurrent repair (i.e. their initial operation was undertaken before the start of the data capture period). A total of 88,590 patients were therefore included. There were 82,917 male patients (93.6%) and 5673 females (6.4%). Median follow-up was 9.8 years (interquartile range 5.7 to 14.6 years). There were 22,184 deaths (25.1%) during the follow-up period occurring at a median of 6.7 years after initial operation (interquartile range 3.4–10.8 years). There were 10,145 laparoscopic cases (11.5%) and 78,445 open cases. Of the laparoscopic cases, there were 210 conversions to open procedures (2.1%). Figure 1 demonstrates a flow chart of the patient cohort and follow-up.

Table 1 shows the demographic and outcome differences of the cohort by operative technique. As a proportion, more bilateral hernias (43.8%) were repaired laparoscopically than unilateral (8.9%). Patients who had laparoscopic repairs were younger and had a shorter median follow-up. The number of laparoscopic cases has increased over time. Patients from more affluent areas were more likely to have had laparoscopic surgery.

**Table 1:** Baseline characteristics

	Open repair		Laparoscopic repair	
Number of patients in group	78,445		10,145	
Demographics				
Male, *n* (% in group)	73,314	(93.5)	9603	(94.7)
Age; years, median (IQR)	60	(46–71)	57	(45–68)
Socioeconomic deprivation status				
SIMD quintile 1 (most deprived), *n* (% in group)	14,139	(18.0)	1437	(14.2)
SIMD quintile 2, *n* (% in group)	15,656	(20.0)	1926	(19.0)
SIMD quintile 3, *n* (% in group)	16,817	(21.4)	1941	(19.1)
SIMD quintile 4, *n* (% in group)	16,943	(21.6)	2074	(20.4)
SIMD quintile 5 (least deprived), *n* (% in group)	14,753	(18.8)	2751	(27.1)
Operative details				
Unilateral hernia repair, *n* (% in group)	74,830	(95.1)	7329	(72.2)
Bilateral hernia repair, *n* (% in group)	3614	(4.6)	2816	(27.7)
Conversion to open procedure, *n* (% in group)	210	(0.3)	n/a	n/a
Year procedure performed				
1996–1999 *n* (% of total in period)	17,631	(95.0)	934	(5.0)
2000–2004, *n* (% of total in period)	21,070	(94.5)	1230	(5.5)
2005–2009, *n* (% of total in period)	21,233	(87.9)	2922	(12.1)
2010–2014, *n* (% of total in period)	18,511	(78.5)	5059	(21.5)
Follow-up				
Median follow-up; years, median (IQR)	10.2	(5.9–15)	7.2	(4.7–10.6)
Events				
No operation for recurrence	51,751	(66.0)	8 021	(79.1)
Operation for recurrence	1397	(1.8)	362	(3.6)
Contralateral operation	6463	(8.2)	495	(4.9)
Died during follow-up	18 834	(24.0)	1267	(12.5)
Median time to death; years, median (IQR)	6.7	(3.4–10.9)	5.9	(3.2–9.9)

### Operation for recurrence: time-to-event analyses

There were a total of 1759 operations for recurrence (2.0% of total): 1397 in the open group (1.8%) and 362 (3.6%) in the laparoscopic group (Table 1). The Cox regression model for time to reoperation for recurrence is shown in Table 2. After adjusting for covariates those receiving a primary laparoscopic repair had greater hazard of having a recurrence than those receiving a primary open repair (hazard ratio 1.83, 95% confidence interval 1.61–2.08, *p* < 0.001). Faster time to reoperation for recurrence was also associated with being older (hazard ratio for 1 year increase in age 1.001, 95% confidence interval 1.007–1.013, *p* < 0.001), being in a higher socioeconomic category (hazard ratio for 5th quintile (least deprived) compared with 1st (most deprived): 1.18, 95% confidence interval: 1.01–1.38, *p* = 0.04) and having a bilateral index operation (hazard ratio: 2.53, 95% confidence interval: 2.22–2.88, *p* < 0.001). Females (hazard ratio: 0.42, 95% confidence interval 0.31–0.56, *p* < 0.001) and those with index operations later in the study period (hazard ratio for one year increase: 0.98, 95% confidence interval: 0.97–0.98, *p* < 0.001) had a lower hazard of reoperation.

**Table 2:** Cox regression models predicting time to a) recurrence, b) mortality, c) non recurrent operation (presumed contralateral)

Dependent variable	Recurrence		Mortality		Contralateral operation	
	HR (95% CI)	*p* value	HR (95% CI)	*p* value	HR (95% CI)	*p* value
Type of operation						
Open	Referent		Referent		Referent	
Laparoscopic	1.83 (1.61, 2.08)	< 0.001	0.87 (0.82, 0.92)	< 0.001	0.85 (0.78, 0.94)	0.001
Age	1.01 (1.00, 1.01)	< 0.001	1.11 (1.11, 1.11)	< 0.001	1.01 (1.01, 1.00)	< 0.001
Gender						
Male	Referent		Referent		Referent	
Female	0.42 (0.31, 0.56)	< 0.001	0.83 (0.79, 0.88)	< 0.001	0.47 (0.41, 0.53)	< 0.001
Socioeconomic deprivation						
SIMD quintile 1	Referent		Referent		Referent	
SIMD quintile 2	1.11 (0.95, 1.3)	0.194	0.85 (0.82, 0.88)	< 0.001	0.98 (0.91, 1.06)	0.684
SIMD quintile 3	1.06 (0.87, 1.25)	0.43	0.74 (0.71, 0.77)	< 0.001	0.96 (0.91, 1.04)	0.426
SIMD quintile 4	1.19 (1.02, 1.39)	0.032	0.67 (0.64, 0.70)	< 0.001	1.03 (0.96, 1.12)	0.362
SIMD quintile 5	1.18 (1.01, 1.38)	0.036	0.61 (0.58, 0.64)	< 0.001	1.02 (0.95, 1.1)	0.485
Bilateral primary procedure^a^	2.53 (2.19, 2.88)	< 0.001	1.04 (0.98, 1.10)	0.197	0.64 (0.57, 0.73)	< 0.001
Year of index procedure*	0.98 (0.97, 0.98)	< 0.001	0.97 (0.97, 0.98)	< 0.001	0.96 (0.95, 0.96)	< 0.001

*Since 1996 (the referent year)

^a^Referent is unilateral repair

As a greater proportion of those undergoing laparoscopic surgery had bilateral hernia repair, a sensitivity analysis excluding primary bilateral hernias was also conducted. The results were similar to the main study results (Table 3). On subgroup analysis by gender, laparoscopy had a higher rate of recurrence in both males (*p* < 0.001) and females (*p* = 0.048).

**Table 3:** Cox regression models predicting time to recurrence for a) bilateral hernias and b) unilateral hernia repairs

Dependent variable	Bilateral hernia repair		Unilateral hernia repair	
	HR (95% CI)	*p* value	HR (95% CI)	*p* value
Type of operation				
Open	Referent		Referent	
Laparoscopic	1.76 (1.39, 2.23)	< 0.001	1.85 (1.58, 2.16)	< 0.001
Age	1.01 (1.00, 1.014)	0.096	1.01 (1.01, 1.01)	< 0.001
Gender				
Male	Referent		Referent	
Female	0.21 (0.05, 0.86)	< 0.001	0.44 (0.33, 0.59)	< 0.001
Socioeconomic deprivation				
SIMD quintile 1	Referent		Referent	
SIMD quintile 2	1.43 (0.94, 2.16)	0.094	1.06 (0.89, 1.26)	0.500
SIMD quintile 3	1.24 (0.81, 1.88)	0.321	1.04 (0.88, 1.24)	0.654
SIMD quintile 4	1.45 (0.98, 2.16)	0.066	1.14 (0.96, 1.35)	0.127
SIMD quintile 5	1.33 (0.89, 1.98)	0.166	1.16 (0.98, 1.38)	0.08
Year of index procedure*	0.97 (0.95, 0.99)	0.008	0.97 (0.97, 0.98)	< 0.001

^*^Since 1996 (the referent year)

### Cumulative incidence functions for recurrence, death and re-operation

The main analysis treated deaths and non-recurrent (primary, contralateral) operations as censored events. Table 2 shows the corresponding models predicting these events and treating the other two events as censored. Those with a primary open operation had greater hazard of dying (hazard ratio 0.87, 95% confidence interval: 0.82–0.92, *p* < 0.001) and of undergoing a primary operation on the other side (hazard ratio: 0.85, 95% confidence interval: 0.78–0.94, *p* = 0.001), even after adjusting for age, gender, SIMD quintile, bilateral status and year of operation.

**Fig. 2:**
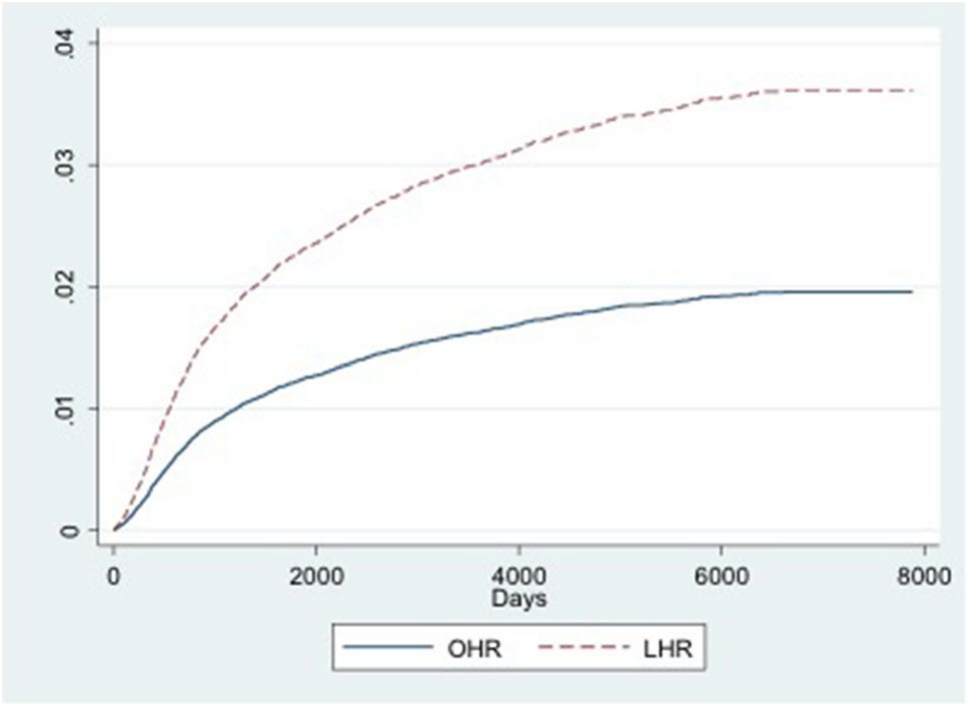
Cumulative incidence function (CIF) for recurrence, by type of index operation. *OHR* open hernia repair, *LHR* laparoscopic hernia repair

**Fig. 3:**
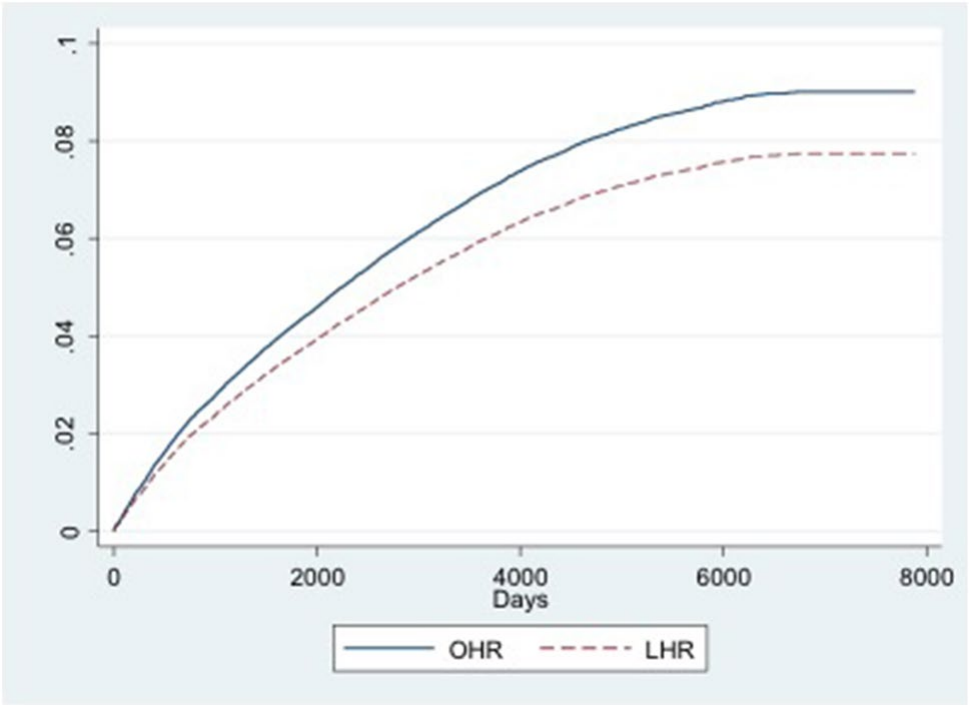
Cumulative incidence function (CIF) for mortality, by type of index operation. *OHR* open hernia repair, *LHR* laparoscopic hernia repair

**Fig. 4:**
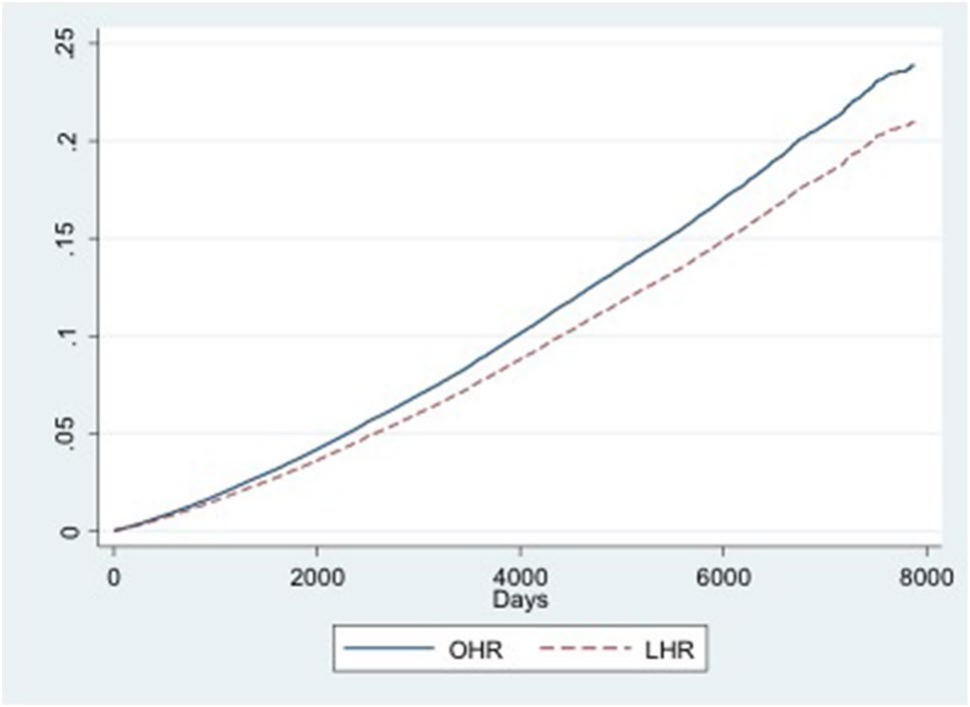
Cumulative incidence function (CIF) for having non-recurrent (primary contralateral) operation, by type of index operation. *OHR* open hernia repair, *LHR* laparoscopic hernia repair

Figures 2,3,4 show the cumulative incidence functions for the three events (recurrence, mortality, and primary contralateral repair). These confirm that the laparoscopic group was more likely to require reoperation for recurrence, but were less likely to die or to have a contralateral repair.

## Discussion

This longitudinal cohort study is one of the largest of its kind. The inclusion of more than 88000 primary inguinal hernia repairs, followed up for a median of nearly 10 years, allows a truly population-wide description of recurrence rates in Scotland. By adopting this study design, the rates of repeat operation after primary hernia surgery in the “real-life” setting, outwith the context of a controlled trial were observed.

The increase in the uptake of laparoscopic hernia surgery amongst Scottish surgeons is clearly demonstrated in this study. More than a fifth of cases in the last 5 years of this study were performed laparoscopically compared to one in twenty in the first 5 years of the study. However, most cases remain open repairs. Laparoscopic repair was associated with an increased risk of requiring surgery for a repair to a recurrent hernia. A faster time to requiring an operation for recurrence in laparoscopic repairs when compared to open surgery is observed, a finding noted even after controlling for factors that might be related to the choice of index operation such as patient age, gender, deprivation category and bilateral hernia status.

Inter-dependent confounding factors influencing absolute outcome are increasingly recognised in the surgical literature as potentially affecting survival analysis [32–34]. Indeed, the lack of consideration for such confounding variables on outcome has recently been described as one of the three common methodological issues observed in population studies [29]. Patients who have died cannot develop a recurrent hernia. In addition, those individuals with contralateral operations and subsequent unilateral recurrences, by the nature of the data coding in the Scottish data, had to be treated as censored in the further analysis. In performing time-to-event analyses controlling for age, gender, socioeconomic deprivation, bilateral status of the index operation, and the year the procedure was performed, it was possible to determine the recurrence rates of the two procedures whilst controlling for potential influencing factors. The findings of operation rates for recurrence being higher following initial laparoscopic surgery, compared with open, are in keeping with several previous, smaller studies [9, 15, 17, 18], but the scale of the current study, both in terms of the size of the population, and length of follow-up, adds to this body of evidence.

Patients might be selected for a laparoscopic instead of open repair for a variety of reasons. Age, gender, and the presence of bilateral hernias appear to have influenced decision making in our cohort. Obesity, size of hernia, comorbidity and surgical experience are also likely to be contributory factors. Interestingly, patients from the most affluent postcodes in Scotland were more likely to have a laparoscopic repair than those living in the more deprived areas. However, data on co-morbidity or body habitus, both of which could also have influenced the choice of approach were not available. Affluence also was associated with an increased likelihood of recurrent hernia operations.

This study has several strengths. It is one of only a few observational studies to use routinely collected, population-based data. Cohort size and duration of follow-up in this work substantially add to the currently available evidence, and the analysis controlled for a number of important confounders. Furthermore, the competing risks modelling, controlling for confounding variables is a fundamental strength of this work.

This study also has limitations, the most important that it is reliant on the accuracy of coding. However, NHS Scotland uses only professional coders trained to maintain minimum coding standards. However, the data were not collected for the express purpose of hernia recurrence analysis and as such there is a risk of information bias. The current coding is also unable to distinguish between transabdominal pre-peritoneal procedures and totally extraperitoneal procedures. It was therefore possible to only have been able to analyse laparoscopic procedures as a combined group, rather than by subgroup. Another key limitation to this analysis is the inability to assess recurrence rates by expertise of the operating surgeon. Although individualised patient data were available, unique codes for the operating surgeon were inconsistently recorded. Furthermore, it is not possible to determine the frequency of the approach undertaken in the private sector. The differences in the cohort demographics may lead to confounding and differences observed could have been the result of factors other than the operative approach. However, the study size and our analytic approach reduce the potential of this problem.

The risk of recurrence after laparoscopic and open inguinal hernia surgery has been studied in a large number of trials and non-randomised studies. This population-wide cohort study, undertaken in the “real-life” setting, of hernia operations being conducted by unselected general surgeons, has permitted us to analyse the risk of recurrence using a large number of patients, over a prolonged follow-up period. A cumulative incidence function approach to control for risk of contralateral operation and death and controlling for cohort differences was adopted. With this, the risk of needing recurrent hernia surgery is higher following initial laparoscopic operations than open procedures. Reoperations for recurrences also are more likely to be required earlier. These results question the notion that the two techniques have equivalent recurrence rates and support open inguinal hernia surgery as the preferred approach for primary inguinal hernia repair.

## References

[1] Jenkins JT, O'Dwyer PJ (2008). Inguinal hernias. BMJ.

[2] Cheek CM (1997). Inguinal hernia repair: incidence of elective and emergency surgery, readmission and mortality. Int J Epidemiol..

[3] McCormack K, Scott NW, Go PM, Ross S, Grant AM, EU Hernia Trialists Collaboration (2003) Laparoscopic techniques versus open techniques for inguinal hernia repair. McCormack K (ed) Cochrane Database Syst Rev. Wiley, Chichester, pp CD001785 10.1002/14651858.CD001785 PMC8407507 12535413

[4] Lichtenstein IL, Shulman AG, Amid PK, Montllor MM (1989). The tension-free hernioplasty. Am J Surg.

[5] Scott N, Go PMNYH, Graham P, McCormack K, Ross SJ, Grant AM (2001). Open Mesh versus non-Mesh for groin hernia repair. Cochrane Colorectal Cancer Group (ed). Cochrane Database Syst Rev.

[6] Ger R, Monroe K, Duvivier R, Mishrick A (1990). Management of indirect inguinal hernias by laparoscopic closure of the neck of the sac. Am J Surg.

[7] Schultz LS, Graber JN, Pietrafitta J, Hickok DF (1990). Early results with laparoscopic inguinal herniorrhaphy are promising. Clin Laser Mon.

[8] Fitzgibbons RJ, Forse RA (2015). Clinical practice. Groin hernias in adults. N Engl J Med.

[9] Ielpo B, Duran H, Diaz E, Fabra I, Caruso R, Malavé L (2018). A prospective randomized study comparing laparoscopic transabdominal preperitoneal (TAPP) versus Lichtenstein repair for bilateral inguinal hernias. Am J Surg.

[10] Douek M (2003). Prospective randomised controlled trial of laparoscopic versus open inguinal hernia mesh repair: five year follow up. BMJ.

[11] Juul C (1999). Randomized clinical trial of laparoscopic versus open inguinal hernia repair. Br J Surg.

[12] Stoker D (1994). Laparoscopic versus open inguinal hernia repair: randomised prospective trial. Lancet.

[13] Andersson B, Hallén M, Leveau P, Bergenfelz A, Westerdahl J (2003). Laparoscopic extraperitoneal inguinal hernia repair versus open mesh repair: a prospective randomized controlled trial. Surgery.

[14] Trevisonno M, Kaneva P, Watanabe Y, Fried GM, Feldman LS, Andalib A (2015). Current practices of laparoscopic inguinal hernia repair: a population-based analysis. Hernia.

[15] O'Reilly EA, Burke JP, O'Connell PR (2012). A meta-analysis of surgical morbidity and recurrence after laparoscopic and open repair of primary unilateral inguinal hernia. Ann Surg.

[16] El-Dhuwaib Y, Corless D, Emmett C, Deakin M, Slavin J (2012). Laparoscopic versus open repair of inguinal hernia: a longitudinal cohort study. Surg Endosc.

[17] Barbaro A, Kanhere H, Bessell J, Maddern GJ (2017). Laparoscopic extraperitoneal repair versus open inguinal hernia repair: 20-year follow-up of a randomized controlled trial. Hernia.

[18] Neumayer L, Giobbie-Hurder A, Jonasson O, Fitzgibbons R, Dunlop D, Gibbs J (2004). Open mesh versus laparoscopic mesh repair of inguinal hernia. N Engl J Med.

[19] Hallén M, Bergenfelz A, Westerdahl J (2008). Laparoscopic extraperitoneal inguinal hernia repair versus open mesh repair: long-term follow-up of a randomized controlled trial. Surgery.

[20] Kukleta JF (2006). Causes of recurrence in laparoscopic inguinal hernia repair. J Minim Access Surg.

[21] Søndenaa K, Nesvik I, Breivik K, Kørner H (2001). Long-term follow-up of 1059 consecutive primary and recurrent inguinal hernias in a teaching hospital. Eur J Surg.

[22] Poobalan AS, Bruce J, King PM, Chambers WA, Krukowski ZH, Smith WC (2001). Chronic pain and quality of life following open inguinal hernia repair. Br J Surg.

[23] Poobalan AS, Bruce J, Smith WCS, King PM, Krukowski ZH, Chambers WA (2003). A review of chronic pain after inguinal herniorrhaphy. Clin J Pain.

[24] Dedemadi G, Sgourakis G, Radtke A, Dounavis A, Gockel I, Fouzas I (2010). Laparoscopic versus open mesh repair for recurrent inguinal hernia: a meta-analysis of outcomes. Am J Surg.

[25] Langeveld HR, van't Riet M, Weidema WF, Stassen LPS, Steyerberg EW, Lange J (2010). Total extraperitoneal inguinal hernia repair compared with Lichtenstein (the LEVEL-Trial): a randomized controlled trial. Ann Surg.

[26] Sajid MS, Caswell J, Singh KK (2015). Laparoscopic versus open preperitoneal mesh repair of inguinal hernia: an integrated systematic review and meta-analysis of published randomized controlled trials. Indian J Surg.

[27] Memon MA, Cooper NJ, Memon B, Memon MI, Abrams KR (2003). Meta-analysis of randomized clinical trials comparing open and laparoscopic inguinal hernia repair. Br J Surg.

[28] Köckerling F, Bittner R, Kraft B, Hukauf M, Kuthe A, Schug-Pass C (2017). Does surgeon volume matter in the outcome of endoscopic inguinal hernia repair?. Surg Endosc.

[29] Zogg CK, Pawlik TM, Haider A (2018). Three common methodological issues in studies of surgical readmission rates. JAMA Surg.

[30] Noordzij M, Leffondre K, van Stralen KJ, Zoccali C, Dekker FW, Jager KJ (2013). When do we need competing risks methods for survival analysis in nephrology?. Nephrol Dialysis Transplant.

[31] Fine JP, Gray RJ (1999). A proportional hazards model for the subdistribution of a competing risk. J Am Stat Assoc.

[32] Cucchetti A, Sposito C, Pinna AD, Citterio D, Cescon M, s (2017). Competing risk analysis on outcome after hepatic resection of hepatocellular carcinoma in cirrhotic patients. World J Gastroenterol.

[33] Grunkemeier GL, Jin R, Eijkemans MJC, Takkenberg JJM (2007). Actual and actuarial probabilities of competing risks: apples and lemons. Ann Thorac Surg.

[34] Taylor SL, Sen S, Greenhalgh DG, Lawless M, Curri T, Palmieri TL (2015). A competing risk analysis for hospital length of stay in patients with burns. JAMA Surg.

